# Letter to the Editor: ‘Hyperglycaemia in pregnancy: Outcomes and diagnostic accuracy of combined modalities’

**DOI:** 10.1016/j.clinme.2026.100556

**Published:** 2026-01-14

**Authors:** Hawkar A. Nasralla, Shaho F. Ahmed, Fahmi H. Kakamad

**Affiliations:** a Scientific Affairs Department, Smart Health Tower, Madam Mitterrand Street, Sulaymaniyah, Iraq; b College of Medicine, University of Sulaimani, Madam Mitterrand Street, Sulaymaniyah, Iraq; c Kscien Organization for Scientific Research (Middle East Office), Hamdi Street, Sulaymaniyah, Kurdistan, Iraq

**Keywords:** Hyperglycaemia, Gestational diabetes mellitus, HbA₁c, HIV, Antiretroviral therapy

Dear Editor,

We read with great interest the recent article by Manga *et al* on hyperglycaemia in pregnancy and the diagnostic performance of combined fasting plasma glucose (FPG) and glycated haemoglobin (HbA₁c) modalities. The authors are to be commended for addressing this important issue within a South African tertiary-care context, where limited resources often preclude universal oral glucose tolerance testing (OGTT).^1^ However, we would like to raise two methodological concerns that were not fully acknowledged and which may have important implications for interpretation.

First, the study reported that 14.8% of participants were human immunodeficiency virus (HIV) positive, with 13.4% receiving antiretroviral therapy (ART). This demographic is highly relevant in the South African antenatal population, yet no stratified analysis or adjustment of HbA₁c performance by HIV or ART status was presented. This omission is noteworthy, given the established evidence suggesting potential bias in HbA₁c measurements among people living with HIV (PLWH). For instance, in a cross-sectional study of HIV-infected individuals receiving zidovudine (AZT)-based regimens, HbA₁c was shown to underestimate mean glucose concentrations by approximately 0.58% compared with non-AZT regimens, due to alterations in haematocrit and erythrocyte indices.^2^ Similarly, a meta-analysis of sub-Saharan African data found that ART exposure was associated with lower HbA₁c values compared with HIV-negative individuals.^3^ Although a recent UK-based study found no strong independent effect of HIV on HbA₁c when continuous glucose monitoring and OGTT were employed, its population comprised older men with type 2 diabetes rather than pregnant women.^4^ Crucially, none of these data directly reflect pregnant women in a high-HIV-burden African setting. In the mentioned study, failure to adjust or stratify diagnostic accuracy by HIV or ART status could introduce systematic misclassification, potentially under-detecting gestational hyperglycaemia in HIV-positive women or overestimating diagnostic performance among HIV-negative women. Given the proposed diagnostic threshold (HbA₁c ≥ 5.75%) and the reported area under the curve (AUC) of 0.93, this bias may not be negligible.

Second, the authors report a median gestational age at booking of 24 weeks. Conducting diagnostic testing at this stage raises the possibility of misclassifying undiagnosed pre-gestational diabetes mellitus (PGDM) as gestational diabetes mellitus (GDM). Evidence suggests that dysglycaemia identified before 20 weeks’ gestation often reflects pre-existing metabolic dysfunction rather than pregnancy-induced changes. For example, Sweeting *et al* demonstrated that women diagnosed with GDM before 24 weeks exhibit metabolic characteristics more akin to type 2 diabetes, including greater insulin resistance and adiposity.^5^ In the study by Manga *et al.*, the absence of early pregnancy glycaemia or HbA₁c measurements limits the ability to differentiate true gestational from pre-existing hyperglycaemia. Consequently, both the estimated GDM prevalence and comparisons of clinical outcomes (GDM versus overt diabetes) may be affected, thereby constraining external validity.

## Funding

This research did not receive any specific grant from funding agencies in the public, commercial, or not-for-profit sectors.

## CRediT authorship contribution statement

**Hawkar A. Nasralla:** Conceptualization, Writing – original draft. **Shaho F. Ahmed:** Validation, Writing – review & editing. **Fahmi H. Kakamad:** Project administration, Supervision, Writing – review & editing.

## Declaration of competing interest

The authors declare that they have no known competing financial interests or personal relationships that could have appeared to influence the work reported in this paper.
